# Diversity and function of bacterial microbiota in the mosquito holobiont

**DOI:** 10.1186/1756-3305-6-146

**Published:** 2013-05-20

**Authors:** Guillaume Minard, Patrick Mavingui, Claire Valiente Moro

**Affiliations:** 1 UMR CNRS 5557, USC INRA 1364, VetAgro Sup, Ecologie Microbienne, FR41 BioEnvironment and Health, Université de Lyon 1, Villeurbanne F-69622, France

**Keywords:** Mosquito, Microbiome, Symbiont, Extended phenotype, Bacterial community

## Abstract

Mosquitoes (Diptera: Culicidae) have been shown to host diverse bacterial communities that vary depending on the sex of the mosquito, the developmental stage, and ecological factors. Some studies have suggested a potential role of microbiota in the nutritional, developmental and reproductive biology of mosquitoes. Here, we present a review of the diversity and functions of mosquito-associated bacteria across multiple variation factors, emphasizing recent findings. Mosquito microbiota is considered in the context of possible extended phenotypes conferred on the insect hosts that allow niche diversification and rapid adaptive evolution in other insects. These kinds of observations have prompted the recent development of new mosquito control methods based on the use of symbiotically-modified mosquitoes to interfere with pathogen transmission or reduce the host life span and reproduction. New opportunities for exploiting bacterial function for vector control are highlighted.

## Introduction

Sustained relationships between prokaryotes and eukaryotes are known to be an important factor in the evolution and speciation of the interacting partners [1]. The classic example of this evolutionary process is the mitochondrion, an organelle essential for cell metabolism in eukaryotes that derives from a bacterial ancestor [2]. Another example is summed up in the coral probiotic hypothesis proposed by Reshef *et al.* (2006) which posits that corals can adapt to their environment by changing their symbiotic bacteria [3]. The symbiotic relationships between microbiota, whether algae, archaebacteria, eubacteria, protozoa or viruses, and their invertebrate host were shown to contribute to the acquisition of resistance to pathogens or tolerance to abiotic stresses [3,4]. Rosenberg *et al.* (2007) recently proposed the hologenome theory to explain such interactions between higher organisms and microbiota [5]. The hologenome theory is based on the concept that higher organisms are not dissociable from their microbial partners, and so together form a unit of selection in which genes from the interacting partners are pooled for the global function of the holobiont [6].

There are numerous examples of microbes influencing so-called extended phenotypes of different taxa, particularly insects which establish association with microbial communities ranging from parasitism to mutualism [7,8]. Bacterial endosymbionts are now known to play roles in many key insect functions such as nutrition, reproduction, development or protection against enemies [8]. For example, the facultative bacterium *Hamiltonella defensa* makes phytophagous aphids more resistant to parasitic wasps [9], whereas the primary symbiont *Buchnera aphidicola* provides essential amino acids [10]. The bacterium *Wigglesworthia glossinidi* is thought to provide vitamins to the hematophagous tsetse fly, an important vector of African trypanosomiasis or sleeping sickness, and benefits in return from carbon sources and protection from the insect host [11,12].

Mosquitoes, the *Culicidae* family, number more than 3,500 different species with a worldwide distribution [13]. Most species described are in the genera *Aedes*, *Anopheles* and *Culex* including several blood feeding members able to transmit pathogens to humans and animals, a great concern for public health [14]. *Anopheles* mostly transmit parasites such as *Plasmodium,* whereas *Aedes* and *Culex* are responsible for the transmission of arboviruses including Dengue (Flavivirus), Chikungunya (Alphavirus) or Japanese Encephalitis viruses, and filariases such as *Wuchereria bancrofti* or *Onchocerca volvulus*. Despite intensive efforts, many mosquito-borne diseases (MBD) are increasing worldwide, partly due to the lack of effective vaccines against etiological agents, but also due to global changes in human activities, especially international travel and trade, that have expanded the distribution of mosquito species previously confined to particular regions. To face these outbreaks, new control strategies based on manipulation of the mosquito hosts and their microbial partners have been proposed recently [15]. A well-known example of this in action is the use of the endosymbiotic bacterium *Wolbachia*[16,17]. This bacterium has a direct impact on the development of some mosquito species by shortening the insect life span, and has indirect effects interfering with pathogen replication and dissemination that affect the vector transmission ability [18,19].

Other than *Wolbachia*, the interactions between mosquitoes and their associated microbiota have yet to be investigated in depth. Most of the published studies describe bacterial diversity and how it varies according to particular factors. Nevertheless, a common conclusion is that a more comprehensive analysis of symbiotic mosquito interactions is needed at evolutionary and functional levels. Better knowledge of the biological impacts will enable the development of efficient biocontrol approaches for MBD. The present work provides an overview of the diversity of symbiotic bacteria and potential functions in the biology of mosquitoes, and highlights the current and future applications in symbiont-based mosquito control strategies.

### Review

#### I-Bacterial diversity and variation in mosquitoes

Complementary approaches are needed for in-depth analyses of microbial communities in complex ecosystems. Both culture-dependent and culture-independent techniques have been used to explore mosquito microbiota. Some microflora can be cultured by using various isolation procedures and media so that bacterial taxa can be identified [20-31] (for details see Additional file 1). The main difficulty of the culture-dependent approach is in recreating the complex physicochemical environment of the insect body [32]. To overcome this limitation and more thoroughly identify bacteria hosted by mosquito populations, culture-independent methods such as Denaturating Gradient Gel Electrophoresis fingerprints, taxonomic microarrays, and meta-taxogenomics can be used (Additional file 1). For example, such molecular approaches, mainly based on analyzing the sequence of the 16S ribosomal RNA gene (*rrs*), have repeatedly shown the dominance of phylum Proteobacteria in mosquitoes [22-24,28,33-35]. Some bacterial taxa are often under-represented in results of these global methods, but primers targeting a particular region of *rrs* or other house-keeping genes can be designed to specifically test for their prevalence in mosquitoes [28,36,37]. While these methods are partially successful, they do not give complete overviews of the mosquito-associated bacterial populations. High-throughput sequencing methods are now being implemented to reveal the previously underestimated microbial diversity, and how certain factors impact the composition and structure of these bacterial populations during the life cycle of mosquitoes [33-35]. Microbial communities may be influenced by host intrinsic factors (species, developmental stage, tissue tropism and genetics), the dynamics of intra- and inter-specific interactions and environmental factors.

### Host species

**Figure 1:**
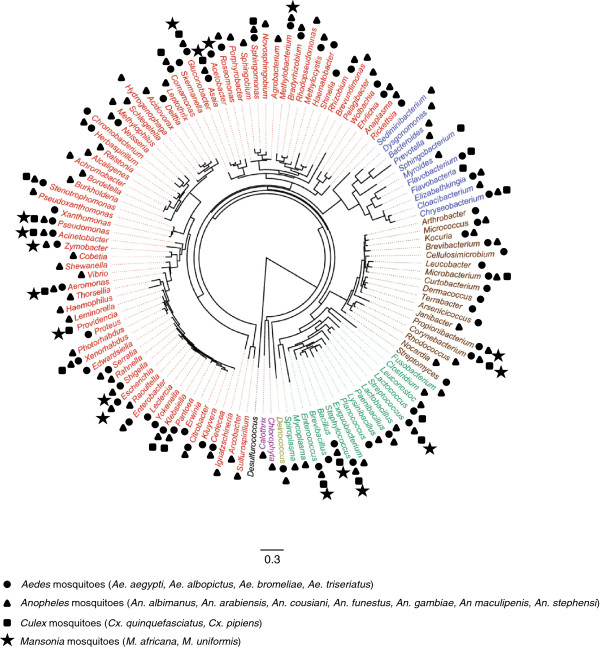
**Bacterial genera identified in *Culicidae*.** Bacteria were classified according to their phyla based on branching in the 16S rRNA sequence phylogenetic tree with names shown in color as follows: Proteobacteria (red), Bacteroidetes (blue), Actinobacteria (brown), [Firmicutes, Tenericutes and Fusobacteria] (green), Cyanobacteria (purple) and Deinococcus-Thermus (yellow). The maximum-likelihood tree was built with an HKY model using 100 bootstraps. A 16S rRNA sequence from *Desulfurococcus* (Archaebacteria) was used as the tree outgroup.

Each mosquito genus has its own preferred habitat and ecological preference. Mosquitoes exhibit particular rhythmic behavioral patterns during their life cycle. For instance, the majority of *Anopheles* and *Culex* species are nighttime biters, whereas some *Aedes* also bite in the daytime. *Anopheles* mostly live in clear water exposed to sunlight whereas *Culex* and *Aedes* are mostly found in dark or troubled water containing a lot of organic matter [38]. In *Culicoides* midges within the same infra order of *Culicidae*, it has been demonstrated that the host species could explain 17% of the variability observed among their bacterial diversity [39]. Surprisingly, there has been no exhaustive comparative study of bacterial diversity across different mosquito species. However, it is possible to compare some surveys and compile the information to highlight specific associations. For instance, when the bacterial content of field populations of adult females of *Anopheles stephensi*, *Anopheles gambiae*, *Aedes aegypti*, *Aedes triseriatus* and *Culex quinquefasciatus* was screened with comparable molecular techniques, it emerged that Proteobacteria was the dominant phylum (Figure 1); notably *Gammaproteobacteria* class representing 41% (for *Cx. quinquefasciatus*) to 86% (for *An. stephensi*) of the total sequences analyzed, while *An. gambiae* hosted mainly *Alphaproteobacteria* and *Betaproteobacteria* classes, possibly because the mosquito specimens were collected at the larval stage and emerged under laboratory conditions [22,24,25,34]. Differences in the proportions of Firmicutes were observed as they account for 13% of sequences analyzed in *Cx. quinquefasciatus*, only 1% in *An. stephensi* and were not detected at all in *Ae. aegypti*. Despite these variations, the core bacterial genome present in mosquitoes seems to be similar in different species. Some genera such as *Pantoea*, *Acinetobacter* or *Asaia* are very prevalent in mosquitoes and capable of cross-colonizing different species [30,40,41].

### Localization in insect host

Bacteria colonize different organs in mosquitoes, mainly the midgut and to a lesser extent salivary glands and reproductive organs [22,24,25,28,42,43]. As in most animal models, the insect gut is a key organ for nutrition and is now considered as being immune-competent [24,32]. The gut is an interface with the external environment and provides resources and space that may be favorable to the multiplication of microorganisms ingested [32]. Active gut bacteria contribute to mosquito digestion through the release of lytic enzymes [44]. In some insect species such as aphids, beetles or cockroaches, specialized structures have evolved for microbial endosymbiosis called bacteriocytes or mycetocytes, which are known to be involved in functions including nutrition and immunity [10,45,46]. None of these structures has been described in mosquitoes. Insect salivary glands, ovaries and hemolymph are also known to be key organs for virus or parasite replication, but surprisingly the bacterial content of these organs in mosquitoes has not been fully characterized. Nevertheless, these organs were specifically screened for some bacterial endosymbionts. For example, the bacterium *Asaia* was detected in salivary glands and reproductive systems of different mosquitoes including *Ae. aegypti*, *An. gambiae* and *An. stephensi*[36,40,47]. The endosymbiont *Wolbachia* was also detected in the head, muscles, Malpighian tubules, ovaries and testes of *Culex pipiens* and *Aedes albopictus*[48,49]. Strikingly, *Wolbachia* was also found in *Ae. albopictus* hemolymph, a fluid which is generally assumed to be bacteria-free [48]. If multiple cell tropisms occur for bacterial partners that are almost fixed in the host population, it is not unreasonable to envisage that a physiological role is yet to be discovered.

### Sex of mosquito

The sex of the mosquito is also an important factor that affects bacterial microbiota composition. Male and female mosquitoes exhibit different ecological behaviors in terms of nutritional and dispersal capabilities. Both sexes feed on nectar and plant saps and are able to hydrolyze sucrose, but females are also hematophagous. Indeed, female mosquitoes are anautogenous as they require blood for the completion of their reproductive cycles [50]. In the mosquito digestion process, different hydrolases are released into the anterior and posterior midgut, which constitutes a selective pressure for resident bacteria [51]. Consequently, the composition and distribution of ingested nutrients themselves may also be a constraint for bacterial communities. For instance, a high concentration of carbohydrates and an acidic pH (from 5.2 to 6.5) occurring in the diverticulum structure are selective for certain bacterial taxa [52,53]. Blood digestion in females is also favored by the selection of bacteria for their hemolytic ability [25,44]. Moreover, after a mosquito ingests a blood meal a temperature burst occurs and oxidative stress and immune responses are down regulated, which leads to an increase in the bacterial load [43,54,55].

**Figure 2:**
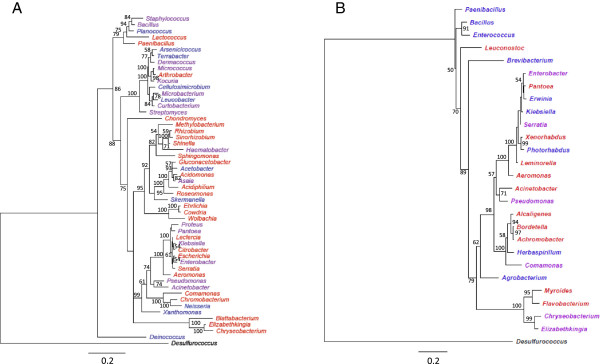
**Phylogenetic dendrograms of bacteria identified in mosquito adults.** Bacterial genera are classified according to mosquito sex of Ae. albopictus (**A**) and An. stephensi (**B**). Names of bacteria identified only in males (blue), only in females (red) or in both males and females (purple) are given. The tree was constructed using the maximum likelihood method with HKY model using 100 bootstraps. Bootstrap values (60% or above) are shown at branch points. *Desulfurococcus* (Archaebacteria) was used as the outgroup.

As mosquito-associated bacteria rely on some of the nutrients brought in the insect meal for growth, the nutrient composition of food sources may directly impact the diversity of bacteria present [24,33]. Zouache *et al.* (2011) showed that around half of the bacterial diversity in field populations of *Ae. albopictus* was explained by the sex of the mosquito with greater diversity observed in females [28]. The effect of the sex of the mosquito on bacterial diversity was also reported in field populations of the malaria vector *An. stephensi*; bacteria from genera *Bacillus* and *Staphylococcus* were detected in males, whereas bacteria from genera *Cryseobacterium, Pseudomonas* and *Serratia* were present exclusively in females [24]. Considering all published data on mosquito-associated bacteria, it appears that the midgut of females is mostly colonized by members of the *Gammaproteobacteria*, as is found in other blood-feeding insects. Interestingly, the genera *Pseudomonas, Serratia* and *Enterobacter* are frequently associated with females of several mosquito species [20-24,26,27,29,30,53]. In contrast, the midgut of males is dominated by bacteria from the phylum Firmicutes including those from *Staphylococcus, Bacillus, Paenibacillus* and *Micrococcus* genera (Figure 2) [24]. Finally, it was also shown that diet, whether sugar or blood meals, significantly affects the bacterial population structure. Wang *et al. (*2011) demonstrated that blood meals drastically reduced the community diversity in favor of enteric bacteria in the *An. gambiae* midgut, while few changes were observed following sugar meals [33]. However, irrespective of the type of meal after 4 days the bacterial microflora reestablishes itself being dominated by the genus *Elizabethkingia*. Finally, male mosquitoes disperse less than females and tend to remain close to breeding sites which could be an additional factor constraining bacterial diversity [50].

### Stages of mosquito development

**Figure 3:**
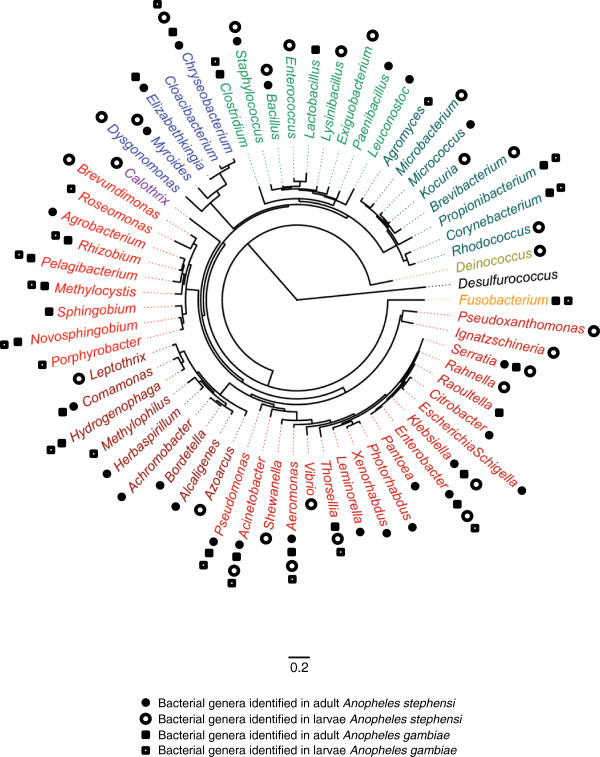
**Phylogenetic dendrogram of bacterial genera identified in *An. stephensi *and *An. gambiae *according to mosquito developmental stage.** Names of bacteria are shown in color as follows: Proteobacteria (red), Bacteroidetes (blue), Actinobacteria, Firmicutes, Tenericutes (green), Cyanobacteria (purple), Fusobacteria (orange) and Deinococcus-Thermus (yellow). The tree was constructed using the maximum likelihood method with HKY model using 100 bootstraps. *Desulfurococcus* (Archaebacteria) was used as the outgroup.

Mosquitoes are holometabola that undergo four gradual stages of metamorphosis - egg, larvae, nymph, and adult - that are intimately connected to their respective biotopes. Eggs, larvae and nymphs are aquatic, whereas adult mosquitoes live in terrestrial environments. The fraction of mosquito-associated microflora that is acquired from the surrounding environment is thus likely to differ during the insect life cycle. At the larval stage, individuals consume bacteria and plankton as nutritive resources. This allows a first stage of bacterial colonization that adds to any inherited bacterial flora. Some of these bacteria such as members of the genus *Wolbachia* are vertically acquired transovarially in *Cx. pipiens*, *Cx. quinquefasciatus* or *Ae. albopictus*. Venereal transmission of the bacterium *Asaia* was reported in *An. gambiae* and *An. stephensi*[47,56,57]. The midgut of mosquito larvae also contains many photosynthetic cyanobacteria acquired from breeding sites which are not found in adults [58,59]. Wang *et al.* (2011) showed that in the larval and pupal stages, cyanobacteria were very abundant accounting for 40% of an entire microbial community in *An. gambiae*[33]. During its metamorphosis, the mosquito anatomy is radically modified. In particular, a first meconial peritrophic matrix or membrane (MPM1) is formed early in the pupal stadium and a second (MPM2) emerged sometimes around the time of adult emergence [60]. A recent study suggests that MPMs contribute to the sterilization of the adult midgut by sequestering microorganisms ingested during the larval stage, which, along with remaining meconial material, are egested after adult emergence [60,61]. This phenomenon could explain why the proportions of different bacterial classes or phyla alter drastically between immature and adult stages. For example, it was shown that the number of bacterial operational taxonomic units (OTU) was 3 fold higher in larvae and pupae than in imagos of *An. gambiae*[33]. To date, comparative studies of bacterial composition between stages have only been done in *Anopheles* mosquitoes, in which transtadial maintenance of some bacterial genera such as *Acinetobacter*, *Bacillus*, *Enterobacter*, *Staphylococcus*, *Pseudomonas*, *Chryseobacterium* and *Serratia* sp. has been observed (Figure 3) [24,26,27,33]. Other mosquito genera should be studied in the same way.

### Ecology

Studies of mosquito-associated bacteria often compare the bacterial communities found in field and lab populations. However, results from lab-reared mosquitoes have revealed the limits of such an approach. By cloning and analyzing signature sequences, Rani *et al.* (2009) demonstrated that the bacterial diversity of midgut microflora in lab-reared *An. stephensi* was less than in field-caught ones, both for males (15 versus 27 bacterial taxa) and females (7 versus 36 bacterial taxa) [24]. Similarly, in *An. gambiae* 45 distinct OTU were identified in lab-reared mosquitoes compared to 155 in field-caught ones using a pyrosequencing approach [34]. Another study of *An. gambiae* also demonstrated that taxa richness in field-caught mosquitoes was higher than in lab-reared ones for any stage and nutritional condition [33]. Bacterial taxa richness in field-caught mosquitoes shows the extent to which bacteria are acquired from the habitat. Environmental factors should be considered as important drivers impacting the load and composition of bacteria in mosquitoes.

As previously discussed, *Culicidae* usually live in highly contrasting environments where biotic (like competition or the food chain) and abiotic (like temperature or humidity) factors can influence their microbiota [38]. The complexity of such ecosystems partly explains some of the conclusions drawn from the few existing studies of the role of environmental factors in modulating bacterial composition in field populations of mosquitoes [24]. Currently, the proportion of bacterial species acquired from the environment is unknown [32]. Each mosquito species has ecological preferences that could determine its bacterial content. For instance, some of the adult microflora is acquired from water during mosquito emergence [23]. Plant and animal hosts are a major source of bacterial acquisition through feeding so have a direct impact on the bacterial colonization of mosquitoes [33]. In *Culicoides sonorensis*, biting midges which transmit viruses to animals, the bacterial flora is derived from soil, plant, bovine and ovine sources [39]. The bacterium *Acinetobacter* was shown to be frequently associated with different mosquito species, including *Ae. aegypti, Ae. albopictus, Ae. triseriatus, An. stephensi, Cx. pipiens, Cx. quinquefasciatus* and *Psorophora columbiae.* Interestingly, this bacterium was also found in mosquito larval breeding sites and in various imago food sources such as vertebrates or plants [20,22,24,28,29,37,62,63]. In a similar way the genera *Asaia* and *Pantoea*, whose natural habitat is the nectar of tropical flowers, were also observed in mosquitoes [41,64,65]. Therefore, the environment may strongly affect the composition of mosquito-associated bacteria. We confirmed this recently by showing that *Ae. albopictus* individuals from urban areas of Madagascar with bush and fruit tree cover differed from those from suburban areas with bamboo cover [28]. We also demonstrated that the prevalence of *Asaia* in *Ae. albopictus* was significantly correlated with the ecological characteristics of sampling sites [37]. Generally, these observations support the idea that field studies are necessary to get an integrated view of mosquito-associated microbiota. However, studies of lab-reared mosquitoes may be a more convenient alternative to evaluate the impact of abiotic factors on the structure and composition of bacterial communities. For example, Wang and coworkers demonstrated that the main bacterial families *Enterobacteriaceae*, *Flavobacteriaceae* and *Pseudomonadaceae* found in lab-reared *An. gambiae* were also identified in field-caught individuals from Kenya [33].

### Interactions between microbial communities

Bacterial interactions are important regulators of ecosystem characteristics and species density. These interactions are ranged along the mutualism to parasitism continuum and structure communities [66]. One interesting example is the human gastrointestinal tract. The gut is naturally protected by a heterogeneous bacterial biofilm, a community of microorganisms living inside an adhesive matrix that forms a mutual structure. Pathogen colonization directly alters (dysbiosis) the biofilm structure [67]. Some recent studies focused on the positive and negative interactions between bacteria inside insect hosts. Terenius *et al.* (2012) tested bacterial interspecies competition with isolates from *Ae. aegypti* and showed that *Serratia marcescens* could create an inhibition zone area on *Sphingomonas* and members of the family *Burkholderiaceae*[30]. The authors suggested a potential link between the presence of *S. marcescens* and the low bacterial diversity observed in the mosquito midgut. Competitive colonization was previously reported in the desert locust *Schistocerca gregaria* where bacterial diversity was shown to increase in the absence of *S. marcescens*[68]. Recently, we found a statistically convincing association between the bacteria *Asaia* and *Acinetobacter* in *Ae. albopictus*[37]. Even though additional analyses are still needed to better understand the degree of interactions between the two genera, we showed that bacterial interaction seems to be synergistic because more *Asaia*-*Acinetobacter* double-infections were observed than would be expected if the bacteria acted independently.

Bacterial symbionts associated with mosquito vectors have recently been found to interact with pathogens they transmit, modifying the outcome of the multipartite interactions. For instance, it was shown that removing bacterial communities from *Anopheles gambiae* increased its susceptibility to *Plasmodium falciparum* infection [69]. On the contrary, Boissière *et al.* (2012) demonstrated that the presence of some bacteria could favor parasite infection, as they found a positive correlation between the abundance of members of the *Enterobacteriaceae* family in the mosquito midgut and the *Plasmodium* infection status [34]. Conversely, Zouache *et al.* (2012) demonstrated that chikungunya virus infection could modify the diversity of symbiotic bacteria in *Ae. albopictus*[34,70]. Indeed, taxonomic microarray and quantitative PCR analyses showed that the abundance of *Enterobacteriaceae* increased with Chikungunya virus infection, whereas the abundance of some other bacterial genera such as *Wolbachia* and *Blattabacterium* decreased [70]. All these results suggest that complex microbial interactions (direct or indirect, cooperation or competition) occur between pathogens and microbiota that may affect mosquito traits such as vector competence.

#### II-Putative impact of bacteria on mosquito biology

The huge bacterial diversity associated with insects and the complexity of potential interactions between symbiotic microorganisms and their hosts pose a significant challenge to understanding extended phenotypes in mosquitoes. Current technologies are not sufficient to pinpoint all the fluxes of matter and energy between microorganisms and their hosts. However, some beneficial functions provided by bacteria, especially those living intracellularly, the endosymbionts, have been deciphered. Generally, insect-associated bacteria are classified in two broad categories, namely primary and secondary symbionts. Primary symbionts or obligate endosymbionts have co-evolved with their insect hosts while secondary symbionts have become associated with their insect hosts more recently and are not obligate. As yet, there is no description of primary endosymbionts in mosquitoes; all studies focusing on secondary symbionts and their potential role in host biology.

### Nutrition

Bacteria contribute to the nutrition of insects in different ways. Midgut bacteria can produce compounds that are directly assimilated by the host or they can improve digestion by producing degradation enzymes which facilitate the assimilation of complex molecules. In phytophagous insects microbiota generally provide vitamins, amino acids and sterol that complement limited plant diets. The best known example is the involvement of the bacterium *Buchnera* in providing essential amino acids to aphids [10]. However, a role for bacteria in nutritional complementation in hematophagous insects has not been demonstrated so unequivocally. One interesting example are the bacteria that provide vitamin B which is not present in vertebrate blood, the sole nutrient source of *Glossina* tsetse flies [71].

In mosquitoes, such a nutritional function has never been formally demonstrated, but some evidence suggests that bacteria could be involved in some processes. For instance, *Serratia* and *Enterobacter*, which are known to contain hemolytic enzymes, could play a role in blood digestion in hematophagous Diptera [25,39,44]. In *Ae. albopictus*, *Acinetobacter baumannii* and *Acinetobacter johnsonii* could be involved in both blood digestion and nectar assimilation [37]. The evidence for this is that unlike environmental *Acinetobacter* strains, mosquito isolates were able to metabolize the amino acids α-keto-valeric acid and glycine, which are blood components, as well as 4-hydroxy-benzoic acid and xylose, which are common constituents of plant sap. The bacterial species *Asaia bogorensis* isolated from *An. stephensi* was shown to be prototrophic with respect to vitamins suggesting it may provide the mosquito with vitamins [40].

Bacteria are involved in nutrition through the release of various compounds useful for mosquito larval development. For instance, it has been demonstrated that a high level of *Pseudomonas aeruginosa* improved larval growth of *Cx. quinquefasciatus* in a phosphorus-rich medium while that of *Cx. tarsalis* was slowed down [72]. The level of phosphorus in breeding sites could be a factor explaining how mosquitoes can adapt to a specific condition according to their bacterial load, possibly with a trade-off between the nutritional and toxic roles of bacteria. Differential tolerance of larvae to putative toxins present in *P. aeruginosa* could explain why the two mosquito species are not found in the same aquatic habitat.

### Reproduction

As previously shown, some bacteria colonize the reproductive organs of insects allowing them to manipulate host reproduction, allowing them to spread considerably through host populations. The genus *Wolbachia* is able to control mosquito mating by a phenomenon called cytoplasmic incompatibility. This process prevents infected males from producing viable progeny when mating with an uninfected female or a female infected with an incompatible *Wolbachia* strain. In this way, certain mosquito species of *Aedes* and *Culex* are dependent on *Wolbachia* to produce viable offspring. Besides *Wolbachia,* other bacteria could play a role in reproduction, such as the genera *Bacillus* and *Staphylococcus* suspected to affect the fertility of the mosquito *Cx. pipiens,* although the mechanisms remain to be determined [73].

### Other potential functions

Bacteria occurring in the environment where mosquitoes mature may also impact on their behavior. This is the case for bacteria producing specific odorant compounds that can act as attractants towards mosquitoes. It was demonstrated that the composition of skin microbiota affects the degree of attractiveness of humans to mosquito species [74]. For example, *Corynebacterium minutissimum produces* volatile compounds *such as* lactic acid or butyl butyrate that attract *An. gambiae*[74]. Moreover, bacteria from breeding sites or water-soluble compounds secreted by those bacteria are able to stimulate the hatching of *Ae. aegypti* eggs [75]. Some studies demonstrated a link between the presence of bacteria in insect hosts and their ability to degrade some insecticide molecules. For instance, the stinkbug which lives on sugarcane may harbor some fenithrotion-resistant *Burkholderia* which are acquired from the environment [76]. The acquisition of these bacteria by each generation could be an easy way for the insect to detoxify itself from the insecticide without any genetic cost. As yet, very few studies have described the role of bacteria in the degradation of xenobiotic molecules, though this could be important in understanding why the number of insecticide-resistant mosquitoes is growing. The load of *Wolbachia* in *Cx. pipiens* seems to be positively correlated with insecticide resistance mediated by esterase genes at some metabolic cost to mosquitoes [77].

Finally, the effects of experimental depletion of the symbiotic strain *Asaia* SF2.1 in *An. stephensi* larvae strongly suggest that the bacterium is a beneficial symbiont of this insect. Indeed, the observation of a delay in the development in larvae after antibiotic treatment in parallel with a dramatic reduction of *Asaia* burden, led to the hypothesis that this bacterium plays a beneficial role in the development of the mosquitoes [78]. Even though the mechanism remains to be identified, the high prevalence of *Asaia* combined with their ability to be transmitted both horizontally and vertically provide evidence of the biological role of bacterium in these mosquitoes [36,47,79].

#### III-Potential applications of bacteria against mosquito vectors

Application of chemical insecticides is still the most common method for mosquito vector control. However, negative consequences like the emergence of insecticide-resistant mosquitoes, environmental contamination and damage to non-target organisms have called chemical-based methods of control into question [80]. The use of bacteria to biologically control mosquito vectors has become a promising strategy.

### Cytoplasmic incompatibility

In the last decade, one of the first efficient strategies to reduce crop insect pests was the introduction of sterile males into a population that, for instance, succeeded in limiting the expansion of the fruit fly *Ceratitis capitata*[81]. The major difficulty of applying the sterile insect technique (SIT) in mosquito populations was the loss of fitness observed in sterilized males [82-84]. Related to SIT, the incompatible insect technique (IIT) was developed based on *Wolbachia*-mediated cytoplasmic incompatibility [85]. The trans-infection of *Ae. albopictus* with *Wolbachia* strains *w*Ri and *w*Pip Istambul originating from *Drosophila simulans* and *Cx. pipiens*, respectively, caused a significant reduction in hatching rates [86,87]. Interestingly, when *Cx. pipiens* was trans-infected with strain *w*Pip Istambul, no impact was observed on the mosquito’s fitness, making this a more promising approach than SIT [82,83,87].

### Paratransgenesis

Transgenesis has also been proposed as a valuable method for controlling mosquito populations. This method is based on the introduction of a transgene in insect vectors which can directly impact on their life history traits or vector competence or indirectly interfere with pathogen replication and transmission [88]. One inconvenience of using transgenic mosquitoes is the cost to mosquito fitness as they are much less competitive [89]. Rather than modifying the insect genome *per se,* a complementary approach called paratransgenesis was proposed, which consists of using a genetically-modified symbiont known to have an impact on insect life history traits [90,91]. Recently, *Asaia* was proposed as a promising symbiotic control agent for paratransgenesis as this bacterium is transformable and can be used to express candidate genes in key organs of infected mosquito species. Similarly, *Pantoea*, a newly identified mosquito symbiont that cross-colonizes several mosquito species and can be transformed and cultured, was also proposed for paratransgenic applications [27]. Recently, transgenic strains of *Pantoea agglomerans* were generated by transformation with a plasmid expressing antiplasmodial compounds [92].

### Modification of vector competence

Vector competence is the ability of a vector to transmit a pathogen, i.e. the intrinsic permissiveness of a vector to be infected, then to replicate and to transmit a pathogen [93]. One strategy used to fight vector-borne pathogens is to decrease vector competence. There is a growing interest in discovering how bacteria interfere with pathogen transmission. In particular, several studies have shown that *Wolbachia* can decrease or inhibit pathogen replication or transmission in different mosquito species. In general, bacteria successfully interfered with pathogens when mosquitoes were trans-infected with strains isolated from a different host. This is the case for *Ae. aegypti* and *Anopheles* which are not naturally infected with *Wolbachia.* In such artificial systems, a significant reduction in life span and pathogen load (including viruses such as Dengue and Chikungunya or parasites such as plasmodiums and filariases) has been observed [16,18,19,94,95]. In *Cx. pipiens* which is naturally infected by *Wolbachia,* the West Nile virus load was reduced only 2–3 fold compared to individuals lacking *Wolbachia*[96]. More recently, the *Ae. albopictus* ALPROV line naturally harboring two *Wolbachia* strains, *w*AlbA and *w*AlbB, was shown to efficiently replicate the dengue virus but transmission, as measured by the amount of genomic RNA and infectious particles in salivary glands, was significantly reduced compared to the *Wolbachia*-uninfected line [97]. Mechanisms of bacterial interference of vector competence still remain to be deciphered, but some hypotheses have been suggested. As bacteria and pathogens can invade similar tissues or even the same cells, a theoretical assumption is that they could directly compete for resources and space [98]. The presence of bacteria could also induce the immune system by producing specific compounds that directly interact with pathogens like antiviral or antiparasitic compounds. Recently, Pan *et al.* (2012) demonstrated that the inhibition of dengue virus in the presence of *Wolbachia* was correlated with the induction of oxidative stress in the mosquito *Ae. aegypti*[99]*.* This response resulted in an activation of the Toll pathway allowing the production of antioxidant molecules and anti-microbial peptides (defensin and cecropins) against dengue virus. In *An. gambiae*, it was shown that oxidative compounds secreted by the strain *Enterobacter* Esp_Z. induced a large decrease in *Plasmodium* in mosquitoes [100]. Joyce *et al.* (2011) showed that half of the bacterial species isolated from *Ae. albopictus* midguts decreased the infectivity of the La Crosse virus in animal cells [101].

### Bacteria used as insecticides

Some bacterial strains are able to produce insecticidal compounds that act like natural pesticides. The bacterium *Bacillus thuringiensis* serovar *israelensis* produces two different toxins encoded by the *cry* and *cyt* genes located on a plasmid replicon [102]. The Cry toxins act on a large insect spectrum (Coleoptera, Lepidoptera, Hymenoptera and Diptera), whereas the Cyt toxins act specifically on Diptera [103]. Both toxins are activated by the alkaline pH of the larval gut and are able to degrade the midgut membrane causing larvae to die [104]. The Firmicute *Lysinibacillus sphaericus* also contains the insecticidal Mtx and Bin toxins that are highly active against mosquito larvae [105]. These toxins paralyze the digestive system and disrupt the insect nervous system. These two classes of larvicidal bacteria are major mosquitocidal candidates and were successfully used in the field to reduce *An. gambiae* populations responsible for malaria outbreaks in Gambia and Ghana [106,107]. Finally, larvicidal toxins of *Clostridium bifermentans* serovar Malaysia and the pupicidal toxin of *Bacillus subtilis* subspecies *subtilis* are also potential candidates as agents in biological control of mosquito populations [108,109].

## Conclusions

Even though information on the nature of mosquito-associated bacteria is increasing, their functions and genetic potential are still underexplored. This is partly due to the complexity of interactions in terms of bacterial population dynamics influenced by different biotic and abiotic factors. In the near future, the application of next generation sequencing should improve our knowledge of the essential microbial partners and their roles in mosquito biology. Interestingly, the recent development of techniques such as metagenomics, metatranscriptomics, metaproteomics and metabolomics is opening up the possibility of more comprehensive descriptions of molecular foundations and signatures of the relationships between insects and their microbiomes. Such high-throughput analysis will allow a better understanding of the dynamics and function of the mosquito-associated microbiota. It will be possible to explore bacterial communities in an unprecedented way by highlighting metabolically active bacteria and discovering novel bacterial genes that play important roles in chemical and biological processes of the insect host. Moreover, with global changes that have greatly contributed to increase the density and geographic expansion of mosquito populations, questions are now raised about the possible scenarios of emergence or re-emergence of mosquito-borne diseases worldwide. As microbial symbionts of insects often mediate or constrain adaptation to environmental fluctuations, better knowledge of mosquito-associated bacterial communities will be an important aspect of understanding what drives mosquito adaptation.

## Competing interests

The authors declare that they have no competing interests.

## Authors’ contributions

GM made the alignments, built the phylogenic trees and drafted the manuscript, CVM and PM helped to draft the manuscript. All authors read and approved the final manuscript.

## Supplementary Material

Additional file 1 **Bacterial genera identified in mosquitoes and their habitats.** An index of bacterial genera identified in mosquitoes and their habitats is presented according to mosquito species, stage, sex, nutrition type, sampling locality, population type and experimental approach (N/A = Not Applicable). Click here for file
